# Effects of acute stress on cue reactivity and implicit cognitions in online compulsive buying-shopping disorder

**DOI:** 10.1556/2006.2025.00002

**Published:** 2025-01-31

**Authors:** Astrid Müller, Maithilee Joshi, Annica Kessling, Nicolas Erdal, Katja Tilk, Christian J. Merz, Oliver T. Wolf, Elisa Wegmann, Matthias Brand

**Affiliations:** 1 Department of Psychosomatic Medicine and Psychotherapy, Hannover Medical School, Hanover, Germany; 2 General Psychology: Cognition, Faculty of Computer Science, University of Duisburg-Essen, Germany; 3 Center for Behavioral Addiction Research (CeBAR), Center for Translational Neuro- and Behavioral Sciences, University Hospital Essen, University of Duisburg-Essen, Germany; 4 Department of Cognitive Psychology, Faculty of Psychology, Institute of Cognitive Neuroscience, Ruhr University Bochum, Bochum, Germany; 5 Erwin L. Hahn Institute for Magnetic Resonance Imaging, Essen, Germany

**Keywords:** craving, attention, implicit associations, stress, internet-use disorder, compulsive buying

## Abstract

**Background and aims:**

There is a lack of research on the impact of acute stress on the interaction of affective and cognitive processes in online compulsive buying-shopping disorder (CBSD). Therefore, this project addressed stress response, cue reactivity, attentional bias, and implicit associations in individuals with online CBSD.

**Methods:**

Women with CBSD (*n* = 63) and women with non-problematic online buying-shopping (*n* = 64) were randomly assigned to the Trier Social Stress Test or a non-stress condition. After the stress/non-stress induction, participants performed a cue-reactivity paradigm, a dot-probe paradigm, and an implicit association task, each with addiction-related (online buying-shopping) and control (social networks) cues.

**Results:**

Individuals with CBSD showed stronger affective responses towards the addiction-related and control cues than the control group and rated the addiction-related pictures with higher ‘arousal’ and ‘urge’ than the control images. No group differences emerged in the dot-probe paradigm and implicit association task. Acute stress showed no effect on performance in the behavioural tasks. Regression models investigating the impact of craving on the relationship between stress response and implicit cognitions within the group with CBSD were not significant.

**Discussion:**

The findings demonstrate the involvement and generalization of cue reactivity in online CBSD, but do not provide support for effects of acute stress on cue reactivity, attentional bias and implicit associations.

**Conclusions:**

Future studies should not be restricted to women and combine laboratory and naturalistic study designs to investigate the complex psychological mechanisms in online CBSD.

## Introduction

Compulsive buying-shopping disorder (CBSD) is characterized by recurrent dysfunctional buying-shopping-related activities that share many clinical features with other disorders due to addictive behaviours (WHO, 2023), such as urges to engage in the behaviour, carrying out the behaviour to regulate internal states, and unsuccessful efforts to control the behaviour despite the many negative consequences caused by inappropriate, maladaptive shopping and purchasing (Brand et al., 2022, 2024; Müller, Laskowski, Trotzke, et al., 2021). With the growth of the e-commerce marketplace and the development of Web 2.0 technologies, online shopping has become increasingly popular. Individuals afflicted with CBSD have largely transitioned from exclusively offline shopping to predominantly online shopping, or alternatively, utilise both offline and online shopping (Müller et al., 2019; Xu, Unger, Bi, Papastamatelou, & Raab, 2022). The online subtype of CBSD can be understood as an excessive online activity associated with marked functional impairment and/or distress in daily life that represents a specific form of problematic usage of the internet (PUI) (Dell'Osso et al., 2021; Fineberg et al., 2018; Fineberg et al., 2022) or a specific online addictive behaviour (Brand, 2022; Brand, Young, Laier, Wölfling, & Potenza, 2016).

Individuals with CBSD suffer from many adverse consequences (e.g., financial constraints, indebtedness, anxiety, shame, guilt, feelings of inferiority, depression) and impairments in important areas of functioning (e.g., familial discord, job loss) (Black, 2022; Brand et al., 2024; Granero et al., 2016). Some persons with CBSD engage in rule-breaking, fraudulent activities in order to mitigate the negative consequences associated with excessive purchasing (Benson, 2013; Meyer, Laskowski, de Zwaan, & Müller, 2019; Müller, Georgiadou, et al., 2022; Park, Cho, & Seo, 2006). Especially with regard to online CBSD, the frequent and time-consuming online search for consumer goods, discounts and bargains significantly interferes with other leisure activities, obligations and commitments.

In contrast to the relatively high number of studies on phenomenology, prevalence, comorbidity and psychosocial correlates of CBSD (Black, 2022; Maraz, Griffiths, & Demetrovics, 2016), research on interactions between affective and cognitive processes is limited. There is, however, evidence for cue reactivity in CBSD on behavioural as well as neural level, i.e. subjective craving responses to shopping-related cues and activities in the ventral and the dorsal striatum as neural correlates of cue reactivity (Starcke, Antons, Trotzke, & Brand, 2018; Thomas, Joshi, Trotzke, Steins-Loeber, & Müller, 2023; Trotzke, Starcke, Pedersen, & Brand, 2021). Several studies demonstrated specific implicit cognitions, such as attentional bias towards shopping-related stimuli and positive implicit associations, but the evidence is mixed and some studies did not show such implicit cognitions in CBSD altogether (cf. Thomas et al., 2023). For example, Vogel et al. (2019) found no differences between treatment-seeking patients with CBSD and control participants in a dot-probe paradigm (Loeber et al., 2009; MacLeod, Mathews, & Tata, 1986) and an implicit association task (Greenwald, McGhee, & Schwartz, 1998), each with shopping-related visual cues. Trotzke, Müller, Brand, Starcke, and Steins-Loeber (2020) used a test battery including the dot-probe paradigm and implicit association task in a convenience sample and observed no relationship between symptoms of CBSD and task performance. Only after considering potential task order effects, CBSD symptoms were correlated with more attentional bias and with positive implicit cognitions in those participants who had performed the dot-probe paradigm or implicit association task in first order (Trotzke et al., 2020).

Overall, the interplay of craving (e.g., strong subjective urge to shop) with implicit cognitive processes in CBSD is largely understudied (Brand et al., 2019; Kyrios et al., 2018; Thomas et al., 2023). Theoretically, it can be assumed that the repeated experience of gratification through the act of buying-shopping may lead to the assignment of incentive salience to shopping-related cues, resulting in an attentional bias towards such cues, and positive implicit associations with buying-shopping (regardless of debts, family discord, etc.), amplifying craving for it (Brand et al., 2019; Robinson & Berridge, 2000). Many studies, however, did not find any effects of implicit cognitions in CBSD (cf. Thomas et al., 2023). A better insight into implicit cognitions and their interaction with craving is important to improve the understanding of the development and maintenance of CBSD, calling for more research on these key concepts of addictive behaviours.

Another important variable to consider in relation to CBSD is stress, which may act as a trans-diagnostic vulnerability factor or as a trigger for excessive shopping and purchasing (Brand et al., 2019; Lawrence & Elphinstone, 2021; Thomas et al., 2024). Stress induces a neuroendocrine response aimed at restoring homeostasis, resulting in changes in mood and behaviour (McEwen, 2008). Permanent exposure to stress is associated with dysregulation of the sympathetic nervous system and hypothalamic-pituitary-adrenal (HPA) axis, which cause the release of catecholamines and glucocorticoids respectively. The Trier Social Stress Test (TSST; Kirschbaum, Pirke, & Hellhammer, 1993) has been used in many studies to induce acute psychosocial stress in a standardized manner. Individuals who completed the TSST showed an increase in negative affect, rapid response in salivary alpha-amylase (sAA; as marker of the sympathetic nervous system) with peak levels immediately after the stress induction, and later salivary cortisol (sCort) increases (as marker of the HPA axis) (Allen, Kennedy, Cryan, Dinan, & Clarke, 2014). Notably, past research suggests an association between blunted HPA stress response to acute psychosocial stress and adverse health outcomes, e.g., depression, obesity, bulimia and addictions (Carroll, Ginty, Whittaker, Lovallo, & de Rooij, 2017). Individuals with problematic internet use exerted a blunted cortisol response to acute stress (Kaess et al., 2017; Tsumura, Fukuda, & Kanda, 2022), while such an effect was not observed in university students with problematic internet use (Bibbey, Phillips, Ginty, & Carroll, 2015).

From a neuropsychobiological perspective, acute stress may lead to more reactive processing of salient addiction-related stimuli (Shields, Sazma, & Yonelinas, 2016) and may boost seemingly habitual dysfunctional behaviour at the expense of goal-directed, cognitively controlled behaviour (Schwabe, Dickinson, & Wolf, 2011; Schwabe & Wolf, 2011) possibly mediated by cortisol responses (Smeets, van Ruitenbeek, Hartogsveld, & Quaedflieg, 2019). Cross-sectional questionnaire-based studies found a correlation between self-reported stress and symptoms of CBSD, along with higher levels of self-reported stress in individuals with risky buying-shopping or in patients with CBSD as compared to control participants (cf. Thomas et al., 2024). To the best of our knowledge, there is a lack of experimental studies directly investigating the role of acute stress in influencing psychological mechanisms in CBSD.

The current study used the TSST (Kirschbaum et al., 1993) or Placebo-TSST (Het, Rohleder, Schoofs, Kirschbaum, & Wolf, 2009) as a control condition to examine the impact of acute stress on cue reactivity, attentional bias and implicit associations in individuals with CBSD (CBSD group) and participants with non-problematic buying-shopping (control group, CG). Given the rise of e-commerce and the shift from in-store to online shopping (Müller et al., 2019; Xu et al., 2022), the study focused on online CBSD. Representative surveys indicate a female dominance of CBSD (Adamczyk, 2024; Laskowski, Hildebrandt, & Muschalla, 2024; Maraz et al., 2016), and women appear to seek treatment for CBSD more frequently than men (Laskowski et al., 2024; Müller et al., 2023). Therefore, the current project investigated exclusively women.

Based on previous research (e.g., Starcke et al., 2018; Thomas et al., 2023; Thomas et al., 2024; Trotzke, Brand, & Starcke, 2017), it was expected that the current study would provide support for cue reactivity and higher levels of self-reported stress in individuals with CBSD as compared to those with non-problematic buying-shopping. Regarding the role of acute stress on cue reactivity and implicit cognitions, the following specific pre-registered hypotheses were formulated:

1. *Cue reactivity*
  Individuals in the acute stress condition will experience higher affective responses (e.g., higher subjective urge) upon confrontation with the specific addiction-related cues (buying-shopping-related pictures) as compared to the non-stressed individuals. The effects of acute stress on affective responses (e.g., subjective urge) will be stronger in the CBSD group as compared to the affective responses in the CG.

The effects of acute stress on cortisol responses were explored without specific hypotheses because no clear conclusions could be drawn from previous studies.

2. *Implicit cognitions*
  2.1 The effects of acute stress on attentional bias to addiction-related visual cues will be stronger in the CBSD group as compared to the CG.
  2.1 The effects of acute stress on implicit associations in response to addiction-related visual cues with positive associations will be stronger in the CBSD group as compared to the CG.
3. *Interaction effect*
  Effects of acute stress versus non-stress on implicit cognitive processes within the CBSD group will be moderated by craving responses. This interaction is expected to be stronger for buying-shopping-related versus control (social-networks-related) pictures.

## Methods

### Participants

The study included a female subsample of the FOR2974 cohort (Brand et al., 2021). Recruitment took place from October 2021 to October 2023. The individuals with CBSD were primarily recruited from the outpatient clinic for behavioural addictions of the Hannover Medical School and from counselling or treatment facilities for behavioural addictions in Lower Saxony (Germany) or the Duisburg-Essen (Germany) area. Additional recruitment took place via flyers, the use of social media platforms, and through personal recommendations. Control participants with non-problematic online shopping habits were recruited through a variety of channels, including advertisements, mailing lists, social networks, and word-of-mouth recommendations. Participants had to be between the ages of 18–65 years and possess sufficient German language skills. Along with these inclusion criteria, individuals in the CBSD group needed to have a clinically relevant case of CBSD as indicated by clinical interview (see below). Control participants should engage in non-problematic buying and shopping activities, without any functional impairment. Exclusion criteria for the entire sample were prior participation in the TSST, learning or developmental disorders, mania, psychosis, suicidal ideations, current substance use disorders, and any medical illness or intake of any substances that interfere with cognitive performance or cortisol response. In addition, body mass index below 17 kg/m^2^, being pregnant, breastfeeding or menstruating, and vaccinations in the week before the experiment and working night shifts the day(s) before laboratory session were exclusion criteria. Participants could take antidepressants if they have been on a stable dose for at least three months.

A power analysis using GPower (version 3.1.9.2) indicated that a total sample size of *N* = 128 (*n*_CBSD_ = 64, *n*_CG_ = 64) is necessary to detect a medium-sized effect of the stress induction (*n*_CBSD_ = 32, *n*_CG_ = 32) compared to the non-stress condition (*n*_CBSD_ = 32, *n*_CG_ = 32) with a power of 0.80. The expected medium-sized effect was based on previous research investigating cue reactivity and implicit cognitions (Brevers et al., 2013; Snagowski, Wegmann, Pekal, Laier, & Brand, 2015; Trotzke, Starcke, Müller, & Brand, 2015, 2019; Vogel et al., 2019). Data of one woman from the CBSD group were dropped from the analyses, because, unexpectedly, she had never used a computer mouse before and completed the questionnaires and behavioural tasks extremely slowly.

### Procedure

**Fig. 1.**
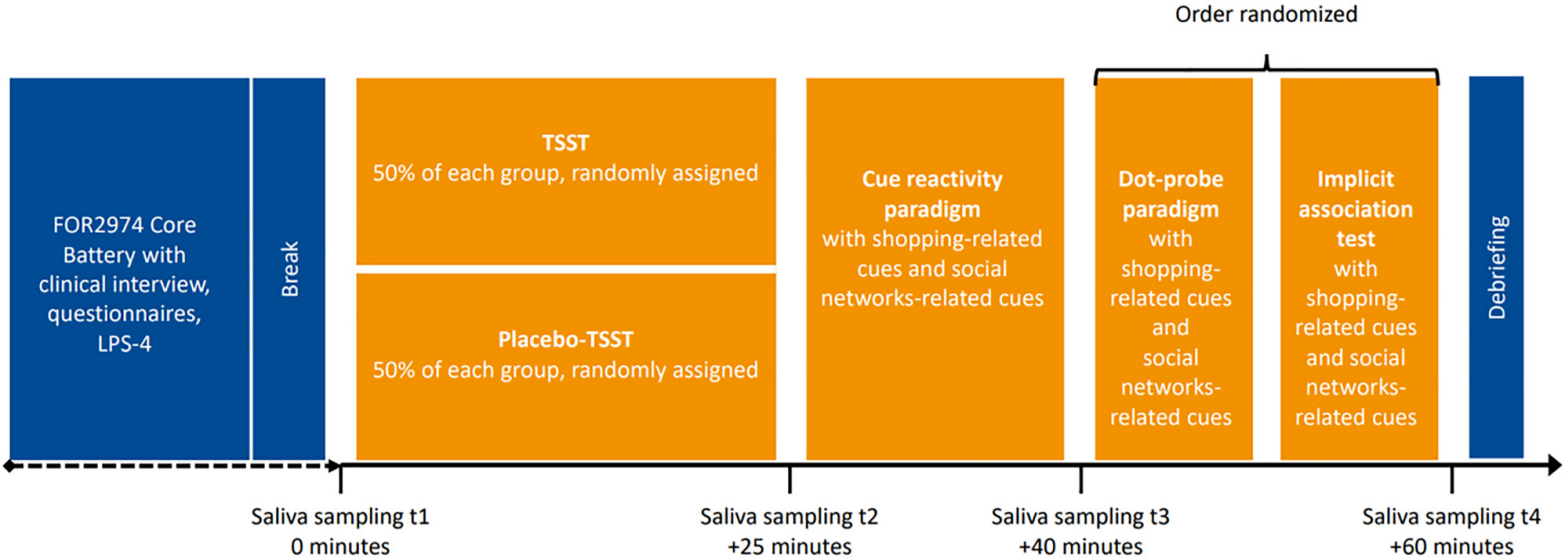
Study design LPS-4 = subtest 4 of the German Leistungsprüfungssystem, TSST = Trier Social Stress Test

The present project is part of the Research Unit FOR2974 (Brand et al., 2021) with specific focus on online CBSD. Telephone screenings were conducted to inform potential participants about the study and to clarify inclusion and exclusion criteria. Eligible individuals were invited to the laboratory session on site, which consisted of two parts and started between 8am and 10am (see Fig. 1). First, participants completed the comprehensive core battery, which was applied in each of the FOR2974 projects (for description see Brand et al., 2021). In this project, the clinical interview, specific questionnaires and the subtest 4 of the German Leistungsprüfungssystem (Horn, 1982) from the core battery were used (described below). Second, the sub-project specific assessments were conducted in the afternoon. Participants of each group (CBSD group, CG) were allocated on a one-to-one basis to either the TSST or Placebo-TSST condition. A fixed simple allocation randomisation was employed without the use of any software. After the TSST/Placebo-TSST, all participants performed a cue-reactivity paradigm and then the dot-probe paradigm and implicit association task. To avoid sequence effects, the two tasks were administered in a randomized order. Saliva samples (Sarstedt, Nümbrecht, Germany) were collected to determine sAA and sCort before (t1; 0 min) and after the TSST/Placebo-TSST (t2; +25 min), as well as after the cue-reactivity paradigm (t3; +40 min) and after the dot-probe paradigm/implicit association task (t4; +60 min). The samples were stored at −20 °C until assayed in duplicates.

All participants provided signed informed consent prior to the testing and were reimbursed with 10 euros per hour. For pseudonymization of the participant data and to comply with the General Data Protection Regulation of the European Union, we used ALIIAS: Anonymization/pseudonymization with LimeSurvey Integration and two-factor Authentication for Scientific research (Englert et al., 2023).

The present project (osf.io/ehq98) as well as the FOR2974 core battery project (FOR2974/RP1; osf.io/6 × 93n) have been pre-registered in the Open Science Framework. From a subgroup of 37 individuals with CBSD, questionnaire data that did not relate to the research questions of the current study were published elsewhere (Wegmann et al., 2023).

### Instruments

#### Diagnostic interview and questionnaires

The interview for the Assessment of Internet and Computer game Addiction (AICA-SKI:IBS) (Müller, Beutel, & Wölfling, 2017; Müller & Wölfling, 2018) was carried out by trained PhD students (MJ, AK, KT). The instrument is based on the DSM-5 criteria for gaming disorder (APA, 2013), adapted for CBSD: (1) preoccupation, craving, (2) tolerance, (3) symptoms of withdrawal, (4) unsuccessful attempts of abstinence/loss of control, (5) loss of interests in previously enjoyed activities, (6) continued buying-shopping despite negative consequences, (7) buying-shopping to regulate emotions, (8) hiding/deception of the amount of buying-shopping, and (9) jeopardizing important relationships/future perspectives (Müller & Wölfling, 2018). Participants in the CBSD group had to meet at least 5 criteria, those of the CG should meet none or at the most one DSM-5 criterion.

The Assessment of Criteria for Specific Internet-use Disorders (ACSID-11) (Müller, Wegmann, et al., 2022) was used to measure the frequency/intensity of online buying-shopping and social network use (11 items for each; 0 = ‘never/not at all intense’ to 3 = ‘often/intense’) in accordance with the ICD-11 diagnostic criteria for gaming disorder, adapted for CBSD and problematic use of social networks (WHO, 2023): (1) impaired control, (2) increasing priority, (3) continuation/escalation. Two additional items measure (4) functional impairment due to use of the certain application. Symptoms of problematic use of social networks were assessed because login pages of social networks served as control cues in the behavioural tasks (see description below). Cronbach's alphas in the present sample indicate good internal consistency of the ACSID-11 frequency scales (buying-shopping, α_CBSD_ = 0.88, α_CG_ = 0.94; social network use, α_CBSD_ = 0.90, α_CG_ = 0.89) and intensity scales (buying-shopping, α_CBSD_ = 0.90, α_CG_ = 0.93; social network use, α_CBSD_ = 0.92, α_CG_ = 0.87). The item scores were dichotomized per criterion and an overall sum score was built (ranging from 0 to 4) as described by Oelker, Rumpf, Brand, and Müller (2024), indicating the number of fulfilled ICD-11 criteria (WHO, 2023).

The Pathological Buying Screener (PBS) (Müller, Trotzke, Mitchell, de Zwaan, & Brand, 2015) was applied to assess symptoms of CBSD regardless of the shopping environment (i.e. offline, online, mixed). The PBS total score consists of 13 items (α_CBSD_ = 0.90, α_CG_ = 0.90) answered on a 5-point Likert scale from 1 (‘never’) to 5 (‘very frequently’).

Given the strong link between CBSD and materialism (Müller et al., 2014; Otero-Lopez, 2022), the groups were further characterized by means of the short Material Values Scale (MVS) (Müller et al., 2013; Richins, 2004). The MVS consists of 15 items (1 = ‘not true’ to 5 = ‘completely true’) that amount to the MVS total score (α_CBSD_ = 0.83, α_CG_ = 0.83).

The overall sum score of the Trier Inventory for Chronic Stress (TICS; Schulz, Schlotz, & Becker, 2004) was utilized to compare self-reported chronic stress between the groups (57 items; 0 = ‘never’ to 4 = ‘very often’; α_CBSD_ = 0.94, α_CG_ = 0.95).

For all measures, higher scores reflect higher (symptom) severity.

The subtest 4 of the German Leistungsprüfungssystem (LPS-4) (Horn, 1982) was used to control the findings of the dot-probe paradigm and implicit association task for general (not addiction-related) logical reasoning abilities. The LPS-4 consists of 40 sequences of letters/numbers that follow a certain design. Participants must discover the one element in each row that disturbs the logical pattern. The number of correct responses (ranging from 0 to 40) was used as outcome.

#### Stress induction

A modified TSST (Kirschbaum et al., 1993) was performed to induce acute psychosocial stress. Participants had to hold a free speech and perform a mental arithmetic task after a brief preparation time (5 min each) in front of a committee (one woman, one men) consisting of two individuals who acted in a reserved and distant manner. The committee members were not included in any of the other assessments. As a control condition, the Placebo-TSST (Het et al., 2009) was used, which lacks the socio-evaluative components of the TSST; in this condition, the participants had to give a speech and to perform an easier mental arithmetic task while alone in the test room (same duration as the TSST). The magnitude of stress response was assessed using the course of the sAA and sCort values (see Procedure).

#### Behavioral tasks

An addiction-specific cue-reactivity paradigm was conducted using the software Presentation (Neurobehavioral Systems Inc., Berkley, CA, USA). Participants were asked to indicate their preferred gadget for shopping: smartphone, tablet, laptop, or desktop. In accordance with the ‘FOR2974 cue-reactivity paradigm’ (Diers et al., 2023), the task consisted of 20 distal addiction-related cues (pictures of login pages of shopping websites) and 20 control images (pictures of login pages of online social networks). All stimuli were presented in blocks of 10 pictures in a pseudorandomised, balanced manner. The participants had to evaluate each picture with respect to ‘arousal’, ‘urge’ (to shop), and ‘valence’ on a 5-point Likert scale (e.g., 1 = ‘no urge’ to 5 = ‘very strong urge’).

A modified dot-probe paradigm (Loeber et al., 2009; Trotzke et al., 2020; Vogel et al., 2019) with 20 addiction-related (logos of shopping websites) and 20 control (logos of online social networks) cues was performed (detailed description in suppl. material S1). An attentional bias score was calculated by subtracting the mean reaction time to respond to a probe replacing an addiction-related cue (congruent trial) from the mean reaction time to respond to a dot replacing a control cue (incongruent trials). Positive values for the attentional bias score suggest an orientation towards the addiction-related cues.

Participants performed a modified implicit association task (Trotzke et al., 2020; Vogel et al., 2019) with addiction-related (pictures of login pages of shopping websites) and control (pictures of login pages of online social networks) cues (detailed description in suppl. material S1). As suggested by Greenwald et al. (1998), the D2D score was used as dependent variable with higher scores indicating stronger positive associations with addiction-related cues.

### Statistical analyses

Analyses were carried out with IBM SPSS Statistics Version 29.0.1.0 (IBM Corp., Armonk, NY, USA). Between-group (CBSD group, CG) and within-group comparisons were made using independent or paired *t*-tests, or *χ*^2^-tests, as appropriate. Repeated measures analyses of variance (ANOVA) with ‘time’ (t1 to t4) as repeated-measures factor, and ‘group’ (CBSD group, CG) and ‘stress’ (TSST, Placebo-TSST) as between-subjects factors were calculated to test whether the stress response (sAA, sCort) across the four measurement points differed between groups. In case of violation of the sphericity assumption, Greenhouse-Geisser correction was applied to the degrees of freedom. In accordance with the pre-registered analysis plan, the primary endpoint of the current study was the task performance in the cue-reactivity paradigm, dot-probe paradigm, and implicit association task (osf.io/ehq98/). Multivariate analysis of variance (MANOVA) was used with affective responses towards addiction-related cues as dependent variables, and group (CBSD group, CG) and stress condition (TSST, Placebo-TSST) as between-subjects factors. For the dot-probe paradigm and implicit association task, the main effect of group and the group by stress interaction were tested (ANOVA). Subsequent ANCOVA were conducted to control the results for logical reasoning as a marker for fluid intelligence. For the CBSD group, moderated regressions with centralised predictor and moderator variables were performed to test potential 2-way interactions of stress and affective responses on task performance in the dot-probe task and implicit association task.

The significance level was set to *p* < 0.05. Cohen's *d* (*t*-tests), Phi (φ) coefficients (*χ*^2^-tests), partial eta squared (η_p_^2^, ANOVAs) or R-squared (*R*^*2*^, regressions) are reported as effect sizes.

### Ethics

The study procedures were carried out in accordance with the Declaration of Helsinki. The Institutional Review Boards of the Hannover Medical School (8767_BO_S_2019) and the University of Duisburg-Essen (ID: 1911APBM0457) approved the study protocol.

## Results

### Demographic and clinical characteristics

Table 1 displays the group comparisons with respect to demographic and clinical variables. No group differences were found regarding age and partnership status. Participants with CBSD had less school years and were more often employed than the CG.

**Table 1.** Demographic and clinical characteristics of individuals with compulsive buying-shopping disorder (CBSD group) as compared to the control group (CG)

	CBSD group	Control group	Test statistic		Effect size
	*n* = 63	*n* = 64		*p*	
**Demographics**					
Age, *mean (SD)*	32.94 (12.41)	32.86 (13.77)	*t(*125) = 0.03	0.974	│*d* │= 0.01
Partnership Status Single, *n* (%)	30 (47.6)	25 (39.1)	*X*^2^(1) = 0.95	0.331	│Ф│ = 0.09
School years					
<11 years, *n* (%)	26 (41.3%)	9 (14.1)	*X*^2^(1) = 11.77	<0.001	│Ф│ = 0.30
≥11 years, *n* (%)	37 (58.7)	55 (85.9)			
Employment Status					
Student, trainee, apprentice, *n* (%)	18 (28.6)	37 (57.8)	*X*^2^(2) = 11.19	0.004	│V│ = 0.30
Unemployed (e.g. housewife/-men, retired), *n* (%)	10 (15.9)	5 (7.8)			
Employed (part-time, full-time), *n* (%)	35 (55.6)	22 (34.4)			
**Clinical characteristics**					
ACSID-11 shopping, *M (SD)*	1.84 (0.68)	0.24 (0.49)	*t*(125) = 15.59	<0.001	│*d*│ = 2.77
ACSID-11 SNS, *M (SD)*^*1*^	1.44 (0.74)	0.62 (0.55)	*t*(115) = 6.78	<0.001	│*d*│ = 1.25
PBS, *M (SD)*	48.56 (9.67)	20.64 (7.06)	*t*(125) = 18.55	<0.001	│*d*│ = 3.30
MVS, *M (SD)*	45.49 (10.39)	36.03 (9.56)	*t*(125) = 5.34	<0.001	│*d*│ = 0.95
TICS, *M (SD)*	26.48 (7.93)	19.42 (8.98)	*t*(125) = 4.69	<0.001	│*d*│ = 0.83
**General cognitive functions**					
Logical reasoning (LPS-4), *M (SD)*	28.40 (4.30)	30.02 (4.08)	t(125) = 2.17	0.032	│*d*│ = 0.39

ACSID-11 shopping = 11-item Assessment of Criteria for Specific Internet-use Disorders, shopping; ^*1*^ACSID-11 SNS = 11-item Assessment of Criteria for Specific Internet-use Disorders, social network sites, data available from 59 patients and 58 control participants; PBS = Pathological Buying Screener; MVS = Material Values Scale; TICS = Trier Inventory for Chronic Stress; LPS-4 = Leistungsprüfsystem, subtest 4.

All participants reported using shopping applications regularly in the ACSID-11 (Müller, Georgiadou, et al., 2022), with higher frequency scores in the CBSD group than the CG. A subgroup of 117 participants (*n*_CBSD_ = 59, *n*_CG_ = 58) reported using online social networks regularly as well. Within the CBSD group, the ACSID-11 means indicate more symptoms of CBSD than of problematic use of social networks (*t*_(58)_ = 3.93, *p* < 0.001, *d* = 0.51). The between-group comparison of ACSID-11 scores suggests more symptoms of problematic use of social networks in the CBSD group than the CG. The CG scored low on both ACSID-11 subscales, with more symptoms of problematic use of social networks than CBSD symptoms (*t*_(57)_ = −6.96, *p* < 0.001, │*d*│ = 0.91).

The groups differed significantly in all other clinical characteristics, with more symptoms of general CBSD (not restricted to online buying-shopping), materialism, and self-reported stress but worse logical reasoning abilities in the CBSD group than in the CG.

### Stress response

**Fig. 2.**
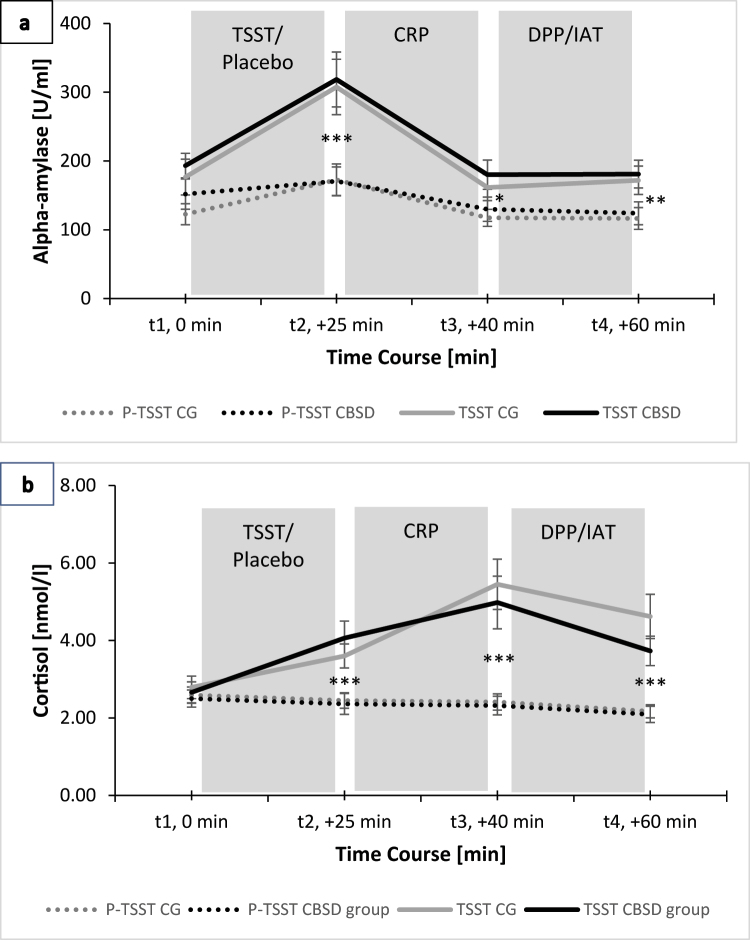
Mean salivary alpha-amylase (2a) and cortisol (2b) responses of participants with compulsive buying-shopping disorder (CBSD group) and the control group (CG) exposed to either the Trier Social Stress Test (TSST) or the Placebo-TSST (P-TSST). CRP = cue-reactivity paradigm, DPP = dot-probe paradigm, IAT = implicit association task Error bars represent standard errors of the mean. ***p < 0.001, **p < 0.01, *p < 0.05 TSST compared to the Placebo-TSST

Figure 2 illustrates the stress responses during the TSST and Placebo-TSST for the CBSD group and the CG. The sAA peak was reached directly after the TSST (Fig. 2a, t2) and the sCort peak about 15 min after completing the TSST (Fig. 2b, t3).

With regard to sAA response, a significant time by stress interaction (*F*(1.68,198.14) = 15.59, *p* < 0.001, η_p_^2^ = 0.12) was found. Pairwise comparisons indicated higher sAA levels at all measurement points in the groups that completed the TSST as compared to the Placebo-TSST, with the greatest difference between the two stress conditions at t2 (see Fig. 2a). There were no significant time by group interaction (*F*(1.68,198.14) = 0.60, *p* = 0.523, η_p_^2^ = 0.01) or time by stress by group interaction (*F F*(1.68,198.14) = 0.33, *p* = 0.678, η_p_^2^ < 0.01).

Considering cortisol responses, we found a significant time by stress interaction (*F*(1.62,196.48) = 19.16, *p* < 0.001, η_p_^2^ = 0.14) with higher cortisol values in the TSST condition as compared to the Placebo-TSST at t2, t3 and t4 (see Fig. 2b). There was no significant time by group interaction (*F*(1.62,196.48) = 1.61, *p* = 0.307, η_p_^2^ = 0.10). Also, no significant time by stress by group interaction was observed (*F*(1.62,196.48) = 1.27, *p* = 0.278, η_p_^2^ = 0.10).

### Cue reactivity

**Fig. 3.**
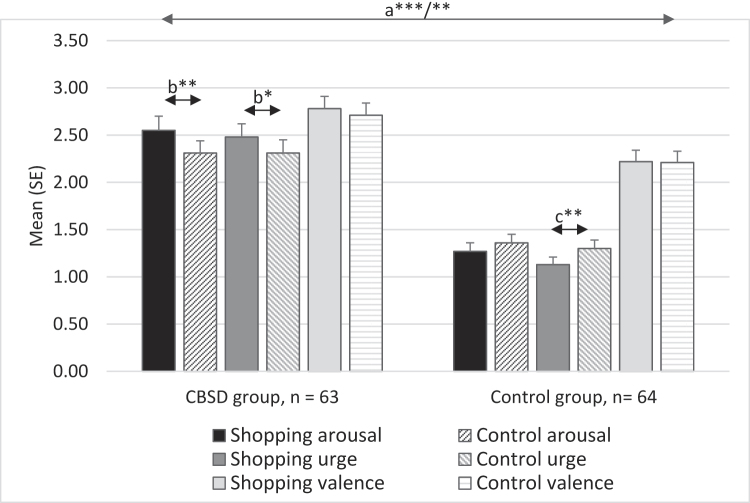
Subjective ratings of visual addiction-related cues (pictures of login pages of shopping websites) and control cues (pictures of login pages of online social networks) for individuals with compulsive buying-shopping disorder (CBSD group) and the control group with non-problematic buying-shopping. a. The CBSD group rated the addiction-related cues (***p < 0.001) and the control cues (**p < 0.01) with higher ‘arousal’, ‘urge’ and ‘valence’ than the control group b. Within the CBSD group, addiction-related cues were rated with higher ‘arousal’ (**p < 0.01) and ‘urge’ (*p < 0.05) than control cues c. Within the control group, control cues were rated with higher ‘urge’ (**p < 0.01) than addiction-related cues.

Figure 3 shows the results of the cue-reactivity paradigm. The CBSD group rated the addiction-related cues with higher ‘arousal’ (*t*_(125)_ = −7.52, *p* < 0.001, │*d*│ = 1.34), ‘urge’ (*t*_(125)_ = −8.31, *p* < 0.001, │*d*│ = 1.47) and ‘valence’ (*t*_(125)_ = −3.18, *p* < 0.001, │*d*│ = 0.56) than the CG. A similar picture emerged for the control cues, with higher ratings in the CBSD group compared to the CG (‘arousal’, *t*_(125)_ = −5.93, *p* < 0.001, *d* = 1.05; ‘urge’, *t*_(125)_ = −6.15, *p* < 0.001, *d* = 1.09; ‘valence’, *t*_(125)_ = −2.80, *p* = 0.003, │*d*│ = 0.50).

Within the CBSD group, addiction-related cues were rated with higher ‘arousal’ (*t*_(62)_ = 3.01, *p* = 0.002, *d* = 0.38) and ‘urge’ (*t*_(62)_ = 1.78, *p* = 0.040, *d* = 0.22) than control cues, but no differences were found for ‘valence’ (*t*_(62)_ = 1.05, *p* = 0.150, *d* = 0.13). The CG rated the control cues with higher ‘urge’ than the addiction-related cues (*t*_(63)_ = −2.56, *p* = 0.006, │*d*│ = 0.32), but did not indicate differences with regard to ‘arousal’ (*t*_(63)_ = −1.54, *p* = 0.064, │*d*│ = 0.19) or ‘valence’ (*t*_(63)_ = −0.04, *p* = 0.482, │*d*│ = 0.01).

Multivariate analysis of variance with affective responses towards addiction-related cues as dependent variables indicate a significant main effect of group (Wilk's lambda 0.58, *F*(3,121) = 28.64, *p* < 0.001, η_p_^2^ = 0.41). With regard to stress, no main effect was found (Wilk's lambda 0.99, *F*(3,121) = 0.49, *p* = 0.692, η_p_^2^ = 0.01). The group by stress interaction did not reach significance (Wilk's lambda 0.94, *F*(3,121) = 2.45, *p* = 0.063, η_p_^2^ = 0.06).

### Attentional bias and implicit associations

Group comparisons of the performance in the dot-probe paradigm and implicit association task are presented in Table 2. With regard to the dot-probe paradigm, the CBSD group showed a higher reaction time in incongruent trials than the CG. However, the attentional bias score did not indicate a significant group difference. Likewise, the groups did not differ in the implicit association task. Given the group differences in the LPS-4 (Horn, 1982) (see Table 1), the analyses were adjusted for logical reasoning as a marker for fluid intelligence, which did not result in group differences either.

**Table 2.** Task performance in the dot-probe paradigm and implicit association task of individuals with compulsive buying-shopping disorder (CBSD group) as compared to the control group (CG)

	CBSD group	Control group	ANOVA			ANCOVA adjusted for logical reasoning^c^		
	(*n* = 63)	(*n* = 64)	*F*(1,125)	*p*	η_p_^2^	*F*(2,124)	*p*	η_p_^2^
*Dot-probe paradigm*								
Attentional bias score, *M (SD)*	−0.02 (21.32)	−1.37 (17.60)	0.15	0.698	<0.01	0.07	0.788	<0.01
Reaction time in congruent trials^a^ [ms], *M (SD)*	348.52 (57.47)	334.63 (38.79)	2.56	0.112	0.02	0.692	0.407	0.01
Reaction time in incongruent trials^b^ [ms], *M (SD)*	348.50 (54.19)	323.06 (71.66)	5.08	0.026	0.04	2.92	0.090	0.02
*Implicit association task*								
D2D, *M(SD)*	0.49 (0.48)	0.33 (0.55)	2.98	0.087	0.02	1.87	0.174	0.02

^a^dotprobe following buying/shopping-specific pictures.

^b^dotprobe following control pictures.

^c^subtest of the German intelligence test battery, Leistungsprüfsystem, LPS-4.

Means and standard deviations presented in the table are unadjusted.

The stress condition (TSST, Placebo-TSST) did not affect the dot-probe paradigm or implicit association task results. No significant group by stress interactions were found for the dot-probe attentional bias score (*F*(1,123) = 1.71, *p* = 0.194, η_p_^2^ = 0.01), reaction time in congruent (*F*(1,123) = 3.73, *p* = 0.056, η_p_^2^ = 0.03) or incongruent trials (F(1,123) = 0.883, *p* = 0.349, η_p_^2^ = 0.01). For the D2D score of the implicit association task, the group by stress interaction was not significant either (*F*(1,123) = 0.621, *p* = 0.432, η_p_^2^ = 0.01).

### Impact of craving on the relationship between stress response and performance in the dot-probe paradigm and implicit association task in individuals with CBSD

Hierarchical moderated regression analyses were computed to test the hypothesis that within the CBSD group the relationship between task performance (dependent variable: dot-probe paradigm or implicit association task) and stress response (baseline-to-peak alpha-amylase/cortisol increase) is influenced by high subjective craving (cue-reactivity paradigm, ‘arousal’, ‘urge’, ‘valence’) towards shopping-related cues. None of the models reached significance (supplementary material, Tables S1 and S2).

## Discussion

The present work supports previous findings regarding the crucial role of cue reactivity in CBSD, as expected (Kyrios et al., 2018; Thomas et al., 2023). The results, however, go beyond previous studies (e.g., Trotzke et al., 2020; Trotzke et al., 2021; Vogel et al., 2019) by showing that individuals with CBSD present with cue reactivity to distal cues. This may be interpreted as generalised cue reactivity [see discussion in Diers et al. (2023), for cue reactivity to distal gaming cues in individuals with risky gaming]. Individuals with CBSD not only show cue reactivity to pictures that involve specific products or buying scenarios (as used in previous studies), but also to starting/login pages without any specific buying content. In addition, the potential effect of generalisation of cue reactivity to stimuli that are only moderately related to buying-shopping activities is also seen in the result that the CBSD group rated the control cues (pictures of login pages of online social networks) with more ‘arousal’, ‘urge’, and ‘valence’ than the CG. Regardless of the clear preference for shopping-related cues as compared to control cues, participants with CBSD appear to have generalized cue-induced craving responses for pictures showing the login pages of online applications. Such login pages may serve as conditioned stimuli that elicit positive affective responses in persons with CBSD, which fits addiction theories (Robinson & Berridge, 2000). It also emphasizes the assumption of stronger and more generalized cue reactivity in later stages of the development of addictive behaviours as proposed by the I-PACE model (Brand et al., 2019). The increasing seamless integration of e-commerce features into social network platforms may have contributed to the result (Han & Kim, 2016; Moghddam, Carlson, Wyllie, & Rahman, 2024; Müller, Laskowski, Wegmann, et al., 2021) of seemingly more generalised cue reactivity in the CBSD group. However, as mentioned above, the shopping-related cues provoked significantly stronger affective responses than the social-network-related cues in the CBSD group. We assume that the choice of control cues was appropriate and, above all, close to the reality of the participants.

With regard to implicit cognitions, no significant differences were observed between the groups. This finding is consistent with those of previous studies that employed the dot-probe paradigm and implicit association task with shopping-related cues to compare individuals with CBSD to those with non-problematic shopping (cf. Thomas et al., 2023). In the implicit association task, we used distal cues (login pages). It is conceivable that proximal cues (e.g., images depicting certain products or a “buy now” button) would have elicited stronger positive associations with shopping. Given the efficacy of the distal cues in the cue-reactivity paradigm, we assume that the outcome should not be attributed to the distal cues. In the dot-probe task, more proximal cues were used (i.e. logos), but this approach also did not yield significant group differences.

At this point, it is important to emphasize that the present study focused on the impact of stress on craving and implicit cognitions in individuals diagnosed with CBSD. Based on the literature on substance use disorders (Schwabe et al., 2011; Schwabe & Wolf, 2011), we hypothesized that the experience of acute psychosocial stress may influence craving responses towards addiction-related cues and alter cognitive processing of emotional information through changing selective attention and implicit associations. Therefore, the participants completed the cue-reactivity paradigm, dot-probe paradigm and implicit association task after a standardized laboratory stress induction (in comparison to a placebo stress condition). The comparison of sAA and sCort levels between the TSST and Placebo-TSST verified the success of the stress induction with the expected rapid response in sAA reactivity and delayed sCort response (Allen et al., 2014), whereas participants with CBSD showed neither a stronger nor an attenuated stress response compared to the CG (see Fig. 2). Opposed to our hypotheses 1 and 2, acute stress did not appear to exert a significant effect on either cue reactivity or implicit cognitive processing. With respect to the dot-probe paradigm and the implicit association task, one potential explanation for the unexpected outcome may be their temporal sequence within the experiment. By the time the dot-probe paradigm and implicit association task were completed, the presumed sCort peak after the TSST was reached and the sAA concentration had already returned to its initial level. It is possible that we would have found an influence of acute stress on task performance if both tests had been performed immediately after the TSST/Placebo-TSST. However, there was also no stress effect in the cue-reactivity paradigm which was carried out directly after the stress induction. A more plausible explanation for the unexpected findings relates to the suitability of stress induction in the laboratory for the investigation of CBSD. The standardised laboratory stress condition may not have sufficiently captured the everyday stressful situations that lead to loss of control over shopping in people with CBSD. Situations involving stress in everyday life may be much more complex and highly dynamic than can be modelled in a laboratory setting. The results addressing hypothesis 3 support this assumption. Although participants with CBSD showed strong affective responses to addiction-related cues (craving) and a significant sAA and sCort stress response in the TSST, the interactions with implicit cognitions were not significant. Again, it remains questionable whether the standardised laboratory design did reflect the proposed stress by CBSD interaction properly. In future studies, this potential methodological shortcoming could be addressed by employing naturalistic approaches, such as the implementation of ecological momentary assessment over an extended period.

Notwithstanding the methodological limitations of the present study, the results to date on affective and cognitive processes in CBSD (Antons, Brand, & Potenza, 2020; Kyrios et al., 2018; Thomas et al., 2023, 2024) give rise to further inquiries. It appears that attentional bias and implicit associations towards shopping-related cues (measured using certain paradigms) are not the primary mechanisms in the development and maintenance of CBSD. It is conceivable that individual differences in other psychological processes may play a more significant role. For example, insufficient stimulus-specific inhibitory control may be a contributing factor. As posited by the inner circle of the I-PACE model, in later stages of the development of addictive behaviours, the imbalance between craving responses and (stimulus-specific) inhibitory control is one of the key mechanisms that may determine whether a specific behaviour is executed in a seemengly habitual manner or not (Brand et al., 2019). The relative lack of experimental studies addressing stimulus-specific inhibitory control in patients with CBSD is surprising (Antons et al., 2020; Kyrios et al., 2018; Thomas et al., 2023). A stop-signal task with shopping-related cues indicated reduced response inhibition abilities in individuals with CBSD compared to control participants in one study (Derbyshire, Chamberlain, Odlaug, Schreiber, & Grant, 2014). However, no difference in task performance was found between individuals with high compulsive buying propensity and those with low compulsive buying propsenity (as measured by questionnaire) in another study (Vogt, Hunger, Pietrowsky, & Gerlach, 2015). Studies using go/no-go tasks with shopping-related versus neutral cues also yielded mixed results. Participants with high symptom scores (as measured by questionnaires) performed worse than control participants in one study (Hague, Kellett, & Sheeran, 2016), while patients with CBSD did not differ from a control group in another study (Vogel et al., 2019). The latter study investigated the potential interactions between craving responses and inhibitory control in both the clinical and the control group. However, no significant interaction effects were identified (Vogel et al., 2019). Taken together, a more thorough examination of stimulus-specific inhibitory control and its interplay with craving in CBSD is warranted.

The present study was restricted to female participants, which represents a limitation. The usual approach in CBSD research of focusing on women is worth criticizing. Even if women are more frequently affected by CBSD and more often seeking treatment than men, this does not mean that CBSD is an exclusively female problem (Laskowski et al., 2024; Maraz et al., 2016). There are possibly sex-/gender-related differences in the psychological mechanisms of CBSD that should be examined not only in survey-based studies as before (Ching, Tang, Wu, & Yan, 2016; Dittmar, Beattie, & Friese, 1995; Mueller et al., 2011; Nicoli de Mattos et al., 2016), but also in experimental research.

Overall, the comprehensive experimental investigation of a well-defined clinical sample with CBSD is an advantage of the present study. Diagnosis of CBSD was confirmed or rejected based on a clinical interview and supported by questionnaire results and clinical features such as higher materialism and general stress levels in the CBSD group as compared to the CG (see Table 1).

## Conclusion

This is the first experimental study that examined the effect of an acute psychosocial stress induction on cue reactivity and implicit cognitions in individuals with addictive use of online shopping applications. While the findings demonstrate the involvement and generalisation of cue reactivity in CBSD, they provide support neither for the proposed attentional bias and positive implicit associations in CBSD nor for the hypothesised effects of acute (laboratory) stress on cue reactivity and implicit cognitions. The combination of laboratory and naturalistic study designs may be a useful approach to gain a more comprehensive understanding of the complex psychological mechanisms contributing to the development and maintenance of CBSD.

## Supplementary material
