# Supplementary material for: Effects of acute stress on cue reactivity and implicit cognitions in online compulsive buying-shopping disorder
Source: J Behav Addict. 2025 Jan 31;14(1):534–47. doi: 10.1556/2006.2025.00002 (PMC11974434; doi:10.1556/2006.2025.00002)
Supplement: Supplementary file 1 [file jba-14-534-s001.pdf]

Müller, A. et al.: Effects of acute stress on cue reactivity and implicit cognitions in online compulsive buying-shopping disorder. https://doi.org/10.1556/2006.2025.00002

Supplementary material S1.

Dot-probe paradigm

In accordance with previous studies (Trotzke et al., 2020; Vogel et al., 2019), a modified dot-probe paradigm (Loeber et al., 2009) with 20 logos of shopping websites (= addiction-related cues) and 20 logos of online social networks (= control cues) was performed. Participants were instructed to indicate the position of a dot (left side or right side) by pressing one of two response buttons on a standard keyboard as fast and accurately as possible. At the beginning of each trial, a central fixation cross was presented for 500 ms, followed by a 200 ms presentation of one addiction-related and one control picture (on the left and right side of the screen, respectively). After picture offset, a white dot probe (Arial, size 50, on a black background screen) was presented, replacing the position of either the addiction-related or the control picture. The dot probe remained until the participant had pressed one of the response keys. Each picture pair was presented four times with counterbalanced side (left/right) and dot probe location (left/right), resulting in 160 presentation trials. Only the pairings with addiction-related pictures were analysed. As dependent variable, the latency of response was recorded (in ms) and trials with response errors and reaction times <100 ms or >1000 ms were excluded. An attentional bias score was calculated for each participant by subtracting the mean latency (ms) to respond to a probe replacing an addiction-related picture (congruent trial) from the mean latency to respond to a dot replacing a control picture (incongruent trials). Positive values for the attentional bias score suggest an orientation towards the addiction-related visual cues.

Implicit association task

In accordance with previous studies (Trotzke et al., 2020; Vogel et al., 2019), a modified version of the implicit association test (Greenwald et al. 1998) was used to assess implicit cognitions towards addiction-related visual cues. With regard to target concepts, this version was modified with pictures

of login pages of shopping websites (= addiction-related cues) and pictures of login pages of online social networks (= control cues). For the attribute concepts, ‘positive’ and ‘negative’ pictures from the International Affective Picture System (IAPS; Lang et al., 2008) were used. Participants were asked to categorize pictures as fast as possible into target concepts (‘buying-shopping’ versus ‘social networks’) and attribute concepts (‘positive’ vs. ‘negative’) by using two differently coloured buttons on a standardized response pad (Cedrus Response Pad RB844, San Pedro, CA, USA). A bipolar structure was selected, since Houben et al. (2010) were able to demonstrate that the choice of ‘positive’ vs. ‘negative’ as attribute concepts outperformed other variants in alcohol-related implicit association tasks (e.g., ‘positive’ vs. ‘neutral’ or ‘negative’ vs. ‘neutral’). In rounds 1 and 2, target (‘addiction-related’ vs. ‘control’) and attribute concepts (‘positive pictures’ vs. ‘negative pictures’) were introduced and practiced. In rounds 3 and 4, target and attribute concepts were combined in an addiction-congruent manner (‘addiction-related or positive’ for one keyboard button versus ‘control or negative’ for the other button). In round 5, the response buttons for the target concept were exchanged and practiced again. In round 6 and 7, target and attribute concepts were combined addiction incongruently (‘control or positive’ vs. ‘addiction-related or negative‘). Within each concept category, ten pictures were presented in a randomized order. It was assumed that individuals with pathological buying-shopping respond faster to congruent pairings (‘addiction-related or positive’ vs. ‘control or negative‘) than to incongruent pairings (‘control or positive’ vs. ‘addiction-related or negative’). As dependent variable, the D2D score was used, which is computed as the difference in reaction times between the incongruent pairings and the congruent pairings divided by their overall standard deviation (Greenwald et al., 2003). Higher D2D scores indicate stronger positive implicit associations with addiction-related pictures. Presentation software (Neurobehavioral Systems Inc., Berkley, CA, USA) was used to present the cues and to record the behavioural responses.

Supplementary table S1. Summary of regression analyses investigating the impact of craving on the relationship between alpha-amylase response and attentional bias (DPP) or implicit associations (IAT) in individuals with compulsive buying-shopping disorder (*n* = 62).

	DPP (attentional bias score)						IAT (D2D)					
	**B**	**SE**	**β**	***t***	***p***	R^2	**B**	**SE**	**β**	***t***	***p***	R^2
sAA response	- .02	.03	- .13	- .74	.465		< .01	< .01	.06	.33	.743	
arousal	.80	2.40	.04	.33	.740		.02	.05	.06	.46	.648	
sAA response x arousal	- .02	.02	- .15	- .86	.395	.02	< .01	< .01	- .19	-1.11	.270	.06
sAA response	- .02	.02	- .14	- .82	.416		< .01	< .01	.02	.12	.902	
urge	.49	2.46	.03	.20	.844		.02	.06	.05	.39	.701	
sAA response x urge	- .02	.02	- .17	-1.00	.322	.02	< .01	< .01	- .26	-1.59	.117	.08
sAA response	- .02	.02	- .13	- .86	.393		< .01	< .01	.08	.53	.595	
valence	- .14	2.63	- .01	- .05	.958		.04	.06	.09	.73	.468	
sAA response x valence	- .03	.03	.20	-1.11	.270	.03	< .01	< .01	- .17	-1.11	.273	.07

sAA = salivary alpha-amylase; DPP = dot-probe paradigm, IAT = implicit association task; sAA resonse = baseline-to-peak sAA increase (i.e. sAA difference t2-t1); arousal, valence, urge to buy = subjective ratings of shopping-related visual cues in the cue reactivity paradigm

Supplementary table S2. Summary of regression analyses investigating the impact of craving on the relationship between cortisol response and attentional bias (DPP) or implicit associations (IAT) in individuals with compulsive buying-shopping disorder (*n* = 63).

	DPP (attentional bias score)						IAT (D2D)					
	**B**	**SE**	**β**	***t***	***p***	R^2	**B**	**SE**	**β**	***t***	***p***	R^2
cortisol response	.40	.91	.06	.44	.664		.01	.02	.05	.36	.720	
arousal	.49	2.40	.03	.20	.840		.02	.05	.04	.31	.761	
cortisol response x arousal	.69	.77	.12	.90	.374	.01	-.03	.02	-.24	-1.77	.081	.07
cortisol response	.45	.92	.07	.49	.625		.01	.02	.05	.37	.715	
urge	.73	2.47	.04	.29	.769		.02	.05	-05	.36	.720	
cortisol response x urge	.79	.82	.13	.96	.338	.02	-.03	.02	-.22	-1.62	.110	.06
cortisol response	.24	.85	.04	.28	.781		.02	.02	.12	.91	.365	
valence	1.09	2.76	.05	.39	.694		.04	.06	.08	.60	.554	
cortisol response x valence	1.74	1.19	.20	1.46	.151	.03	-.01	.03	-.06	-.41	.680	.03

DPP = dot-probe paradigm, IAT = implicit association task; cortisol response = baseline-to-peak cortisol increase (i.e. cortisol difference t3-t1); arousal, valence, urge to buy = subjective ratings of shopping-related visual cues in the cue reactivity paradigm
